# Incidence of Human and Free-Ranging Wild Rodent Infections with *Leishmania* (*Viannia*) *braziliensis*, Aetiological Agent of Cutaneous Leishmaniasis

**DOI:** 10.3390/pathogens12121395

**Published:** 2023-11-28

**Authors:** Orin Courtenay, José F. Marinho-Júnior, Maria Edileuza F. Brito, Juliana F. C. L. S. Monteiro, Jeffrey J. Shaw, Sinval P. Brandão-Filho

**Affiliations:** 1 Zeeman Institute for Systems Biology & Infectious Disease Epidemiology Research, and School of Life Sciences, University of Warwick, Coventry CV8 2PB, UK; 2 Departament of Immunology, Instituto Aggeu Magalhães/FIOCRUZ, Cidade Universitária, Recife 50740-465, PE, Brazil; jrfar8@gmail.com (J.F.M.-J.); edileuza.brito@fiocruz.br (M.E.F.B.); juliana.figueiredo@fiocruz.br (J.F.C.L.S.M.); sinval.brandao@fiocruz.br (S.P.B.-F.); 3 Department of Parasitology, Institute of Biomedical Sciences, University of São Paulo, São Paulo 03001-000, SP, Brazil; jayusp@hotmail.com

**Keywords:** leishmaniasis, *Leishmania* (*Viannia*) *braziliensis*, rodents, skin test, reservoirs, transmission

## Abstract

Background. Human and wild rodent infection rates with *Leishmania* (*Viannia*) *braziliensis* are needed to differentiate transmission pathways in anthropogenically altered habitats. Methods. Human participants in northeast Brazil were tested by the leishmanin skin test (LST) and inspected for lesions/scars characteristic of American clinical leishmaniasis (ACL). Molecular (PCR/qPCR) test records of free-ranging rodents were available from a concurrent capture–mark–recapture study. Force of Infection (λ) and recovery (ρ) rates were estimated from cross-sectional and longitudinal datasets. Results. Cumulative prevalences of human LST+ves and ACL scar+ves were 0.343–0.563 (*n* = 503 participants) and 0.122–0.475 (*n* = 503), respectively. Active ACL lesions were not detected. Annual rates of LST conversions were λ = 0.03–0.15 and ρ = 0.02–0.07. The probability of infection was independent of sex and associated with increasing age in addition to the period of exposure. Rodents (*n* = 596 individuals of 6 species) showed high rates of exclusively asymptomatic infection (λ = 0.222/month) and potential infectiousness to the sand fly vector. Spatially concurrent rodent and household human infection prevalences were correlated. Conclusions. Human exposure to *L.* (*V.*) *braziliensis* continues to be high despite the substantial drop in reported ACL cases in recent years. Spill-over transmission risk to humans from rodents in peridomestic habitats is likely supported by a rodent infection/transmission corridor linking houses, plantations, and the Atlantic Forest.

## 1. Introduction

Concurrent estimates of infection incidence in humans and associated wildlife reservoirs of vector-borne human parasitic diseases are limited, yet it is important to understand their relative role(s) in maintaining zoonotic transmission cycles. One group of diseases of public and veterinary health concern is leishmaniasis, which results from infection with *Leishmania* (Kinetoplastida: Trypanosomatidae) transmitted by Phlebotomine sand flies (Diptera: Psychodidae). Of the polymorphic clinical outcomes, cutaneous leishmaniasis (CL) is the most common, accounting for an estimated 700,000 to 1 million new cases annually [1]. One aetiological agent of American CL (ACL) throughout much of Central and South America is *L.* (*Viannia*) *braziliensis,* for which the clinical prognosis is complex. Following the apparent chemotherapeutic or spontaneous recovery of cutaneous lesions, the parasite can metastasize, causing serious disfigurement through the destruction of the nasal and oral mucosa, pharynx, and larynx, known as mucocutaneous leishmaniasis (MCL).

*L.* (*V*.) *braziliensis* is spread to humans in a zoonotic transmission cycle involving one or more animal reservoirs and sand fly vectors. The number of animal species in which *L.* (*V*.) *braziliensis* has been detected is extensive. However, few studies have attempted to quantify the role of domestic or wild non-human hosts in transmission and in maintaining transmission at epidemiological scale. There is growing consensus that the wildlife reservoir of *L.* (*V*.) *braziliensis* in Brazil is a variable number of small mammal species acting as a collective reservoir guild. This is based on measures of infection prevalence and, for some species, their potential infectiousness to sand fly vectors [2,3,4,5,6]. In the highly endemic region of northeast Brazil, studies of free-ranging small mammal communities identified infection in a multitude of rodent species in intradomicillary, peridomestic, and sylvatic environments [3,4,6]. Of the five rodent species so far tested by xenodiagnosis, all proved capable of transmitting infection to the locally predominant sand fly vector *Nyssomyia whitmani* and to a suspected additional vector, *Lutzomyia longipalpis* [2,6].

Human exposure to *L.* (*V*.) *braziliensis* usually results in a cutaneous lesion at the site of the infectious sand fly bite, but which usually heals spontaneously or following successful treatment within weeks of the lesion’s appearance, leaving a characteristic ACL scar. Recovery from infection and cutaneous disease usually results in humans mounting a delayed-type hypersensitivity (DTH) reaction associated with a Th1 immune response detected by a dermal reaction (induration) to leishmanin (*Leishmania* antigen) administered by the Leishmania skin test (LST). A positive LST response, interpreted as evidence of cell-mediated immunity (CMI), is considered to be protective against reinfection and leishmaniasis disease. LST is a very useful epidemiological tool to investigate *Leishmania* exposure and infection rates, yet few such studies have been conducted in *L.* (*V*.) *braziliensis* endemic regions, and production of leishmanin has been limited over concerns of safety and ethics [7].

The aim of this study was to estimate rates of *L.* (*V*.) *braziliensis* infection in human residents and the local rodent reservoir population in an ACL/MCL endemic region of northeast Brazil. Longitudinal infection and demographic records were available from a 27-month capture–mark–recapture (CMR) prospective cohort study of the free-ranging naturally infected rodent population [6].

## 2. Materials and Methods

### 2.1. Ethical Considerations

Ethical protocols for human testing were approved by the national ethical committee, Comite de Ética em Pesquisa, CEP/CPqAM, Recife, Brazil (number 04/13), and the Biomedical and Scientific Research Ethical Committee (BSREC), University of Warwick, UK (number REGO-2014-1272). Fully informed, signed consent was obtained from adults and, by proxy, from a guardian or parent of children <18 years old. The rodent data were generated under the ethical approvals of the Ethics Committee on Animal Use (CEUA/IAM), Brazil (No. 017/2011), and IBAMA (Brazilian Institute of Environment and Renewable Natural Resources) for Activities of Scientific Purpose (No. 12749) as previously described [6].

### 2.2. Study Site

The study was conducted in three semi-rural/rural communities within the “Zona da Mata” (Atlantic Forest Zone) in the ACL/MCL endemic State of Pernambuco, northeast Brazil, situated <100 km west of the state capital Recife. LST surveys of humans were conducted in the municipalities of Amaraji (Engenhos Raiz de Dentro and Refrigério [8°22′12.5482″ S, 35°27′59.9250″ W]), Moreno [8°6′51.9106″ S, 35°5′26.3584″ W], and Vicência [7°39′37.7125″ S, 35°19′19.0419″ W]. Data from the CMR study of rodents were collected in Amaraji as described [6].

### 2.3. Human Recruitment

Household residents in each of the three municipal foci were invited to the local school to receive information on the purpose of the study and to invite their participation in the study. The sample was a convenience sample, recruiting and testing participants as they arrived at the school over a limited five-working-day period in each foci. Eligibility criteria included (i) individuals who were >2 years old and (ii) normally resident in the locale. Skin testing was conducted by trained health workers in 2014, in February–April in Amaraji, May in Vicência, and September in Moreno.

### 2.4. Leishmanin Skin Test (LST)

Leishmanin skin test antigen was supplied by the Ministry of Health, Brazil, comprising 1 × 10^7^ *L.* (*Leishmania*) *amazonensis* (MHOM/BR/73/PH8) prepared in January 2013 (LOT: 01/2012) by the Controle de Produção e Pesquisas de Immunobiológicos (CPPI, SES-PR). Following standard protocols, 0.1 mL of the antigen solution was injected intradermally on the lower volar aspect of the forearm, and 0.1 mL of control solution containing buffered phosphate saline and 0.01 percent thimerosal was injected 20 cm above the *Leishmania* antigen on the same arm. Readings were taken 48 to 72 h later by trained personnel and measured using the “ball point” method [8]. A mean induration diameter of >5 mm relative to the control was considered LST-positive. No adverse reactions were observed or reported.

### 2.5. ACL Lesions/Scars and Metadata Collection

Participants were examined for active cutaneous or healed lesions (scars) attributed to *Leishmania* infection based on known characteristics by trained clinical inspections. The number, size, and body location of all scars were recorded. Participants were also requested to provide details of their age, sex, period of local residence, and previous location of residence, and to recall the calendar year and their age when a *Leishmania* cutaneous lesion first appeared.

### 2.6. Rodent Trapping and Sampling

The CMR study methods are fully described by Marinho-Júnior et al. [6]. Eight rodent species—between May 2012 and August 2014—were live-trapped in rural communities in Amaraji municipality by setting Tomahawk traps (45 cm × 21 cm × 21 cm) on 9920 single trap nights in the house yard (peridomestic) (620 trap nights), adjacent household plantations (6820), and in pockets of Atlantic Forest (2480) during 20 independent trapping rounds over 27 months. The approximate distances from the peridomestic household trap sites to plantations and Atlantic Forest trap sites were 20–40 m and 100–150 m, respectively.

On capture, rodents were speciated, sexed, and assigned to an age class at first capture (YY—juvenile; JA—young adult; AA—mature adult) based on their body size by experienced field technicians. They were then marked with individual identification microchips, clinically inspected, and a skin biopsy and blood sample collected for detection of *Leishmania* before being released at the site of capture. Individual rodents were (re)captured and sampled 1–11 times each and diagnostically sampled at a median interval of 50 (IQR: 35–91) days. *L.* (*V*.) *braziliensis* parasite presence/absence and parasite loads were quantified by conventional PCR and quantitative PCR (qPCR), respectively. Individuals of five of the rodent species were also xenodiagnosed by exposing them a total of 44 times to the proven local sand fly vector *Ny. whitmani*; the blood-fed flies were screened for *L.* (*V*.) *braziliensis* by qPCR as previously described [6].

Rodents were classified at each (re)capture as infected if samples proved positive for *L.* (*V*.) *braziliensis* by PCR or qPCR.

### 2.7. Rodent Xenodiagnosis

The xenodiagnosis procedures and parasite detection methods are fully described [6]. For the current analyses, 37 individual rodents of five species were xenodiagnosed by exposing them a total of 41 times to unfed females of the local sand fly vector *Ny. whitmani*. Captured rodents were anaesthetised and placed into a Barraud cage, into which sand flies were released and allowed to feed for 1 h. After removal, sand flies were separated and individually stored in a tube containing 70% ethanol for subsequent DNA extraction and qPCR testing. A median of 34 (95% C.L. 19–48) blood-fed female flies were tested from each xenodiagnosis experiment. As the sand flies were killed and preserved immediately after blood-feeding, xenopositive animals are considered here as potentially infectious; it is assumed that a proportion of the detected parasites would develop into transmissible metacyclic promastigotes.

### 2.8. Data Analyses

The period of residency (years) in the endemic study foci reported by participants was considered a surrogate of cumulative exposure to potential infection. For rodents, the interval (days) between the date of first capture and the date of subsequent recapture(s) was used to calculate incidence. For animals classified as juveniles (JJ) at first capture, we assumed an age of 6 weeks for age-incidence calculations.

### 2.9. Infection Estimates

The Force of Infection (FOI) was calculated by fitting the cross-sectional prevalence data to a standard incidence–recovery model

(1) $$p\left(a\right)=\frac{\lambda }{\lambda +\rho }\left({1-e}^{-\left(\lambda +\rho \right)a}\right)$$

where *λ* is the instantaneous incidence, *a* is the estimated age (rodents) or period of resident exposure (humans), and *ρ* is a recovery constant representing the loss rate of infection from the population with cumulative exposure. The model assumes homogenous mixing, a constant force of infection independent of age and time, replacement by susceptible animals, and immediate detection of infection [9]. This model gave a better fit than simple catalytic or variable catalytic models [10].

For rodents, FOI values were also calculated from the longitudinal data, where the number of incident infections *I* in animals uninfected at first capture *S* after a mean follow-up interval *t* is given by

(2) $$\lambda =-ln\left(1-\frac{S}{N}\right)/t$$

### 2.10. Statistical Analyses

Human LST and ACL scar status and rodent infection or infectious status were each analysed as binary dependent variables using binomial complimentary log–log models. To test for potential differences in infection outcomes between physical and demographic attributes (study foci, sex, age, rodent species, habitat type), period of exposure or age as appropriate were forced into all models. To assess differences in periods of exposure between locations or between infection classes, negative binomial models were employed for the detection of significant over-dispersion in shape parameters by goodness-of-fit testing. In secondary analyses, exposure × location interaction terms were included to adjust model outcome estimates. Analyses were performed using STATA v.17 software (StataCorp LP, College Station, TX, USA).

## 3. Results

### 3.1. Humans

A total of 524 individuals with a median age of 20 years (IQR: 5.0–60.0) were recruited from the three study communities (Table 1). LST test results and period of resident exposure were available for 503 participants, and 524 were measured for the presence/absence of *Leishmania* ACL lesions or scars. Data on age or period of exposure/appearance of cutaneous lesions/scars were missing for two individuals. Details of participants’ LST and ACL scar status, age, sex, foci where resident and period of exposure are summarised in Table S1.

### 3.2. LST Infection Rates

The LST+ve prevalence in individual foci ranged between 0.343–0.563 and was 0.455 overall (Table 2). The probability of being LST+ve increased significantly with the reported period of residency in the endemic foci (z = 3.53, *p* < 0.001); the median period of exposure was greater in Vicência than in both Amaraji and Moreno (>3.96, *p* < 0.001), and lower in Moreno than in Amaraji (z = −4.71, *p* < 0.001) (Table 2).

**Figure 1:**
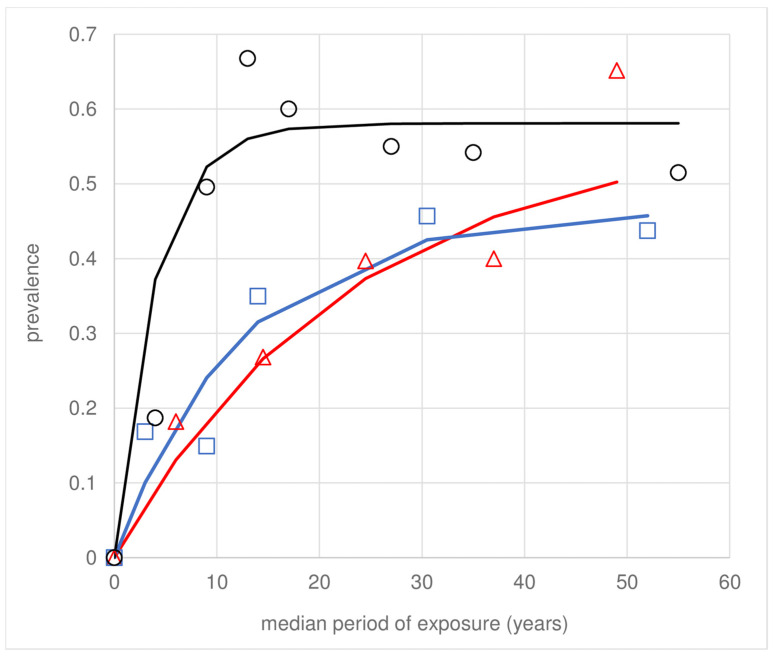
Human LST+ve prevalence with cumulative exposure in the three study foci in Pernambuco State, Brazil: Amaraji (red), Moreno (blue), and Vicência (black). The observed LST prevalence data (symbols) are fitted to the incidence–recovery model (Equation (1)) by maximum likelihood (line fits).

Solving λ and ρ (Equation (1)), the annual mean FOI of LST+ve conversions across the population was λ = 0.07/year, and loss of LST+ves ρ = 0.05/year, with some variation between foci (Table 2). These estimates represent a monotonic initial rise and subsequent dampening of cumulative LST+ve prevalence with increasing exposure (Figure 1); consequently, the asymptotic prevalence does not reach unity: ~0.6 in Vicência and ~0.45 in Moreno. No clear asymptote was observed for Amaraji, which is likely a consequence of the smaller sample size (Table 2). Visual inspection of Figure 1 indicates that the average participant experienced infection in childhood, younger in Vicência than in Moreno or Amaraji, reflecting the higher FOI in Vicência (Table 2).

### 3.3. ACL Scars

No active ACL lesions were recorded at the time of examination, but 31.9% (167/524) of participants presented one or more visual ACL scars: 63.5%, 22.3%, and 14.2% of individuals had 1, 2, and >2 scars (range 3–16), respectively, which presented on all parts of the exposed body (feet, arms, legs, torso, neck, and head). The prevalence of ACL scars in the study communities ranged from 0.214 to 0.475 (Table 3). The risk of presenting an ACL scar increased with the reported period of resident exposure (z = 3.04, *p* = 0.002) and was higher in Vicência than in Amaraji (IRR = 2.4 [95% 1.37, 4.07], z = 3.09, *p* = 0.002) and in Moreno (IRR = 4.3 [2.70, 6.82], z = 6.16, *p* < 0.001). Of the 167 participants with scars, age-recall responses were obtained from 138, and most data (105) were from participants resident in Vicência. Based on these responses, the median age when ACL skin lesions first occurred ranged from 1 to 62 years old (median 14 years) (Table 3), being younger on average in Vicência than in the other two foci (z > 4.63, *p* < 0.001). Age recall of ACL scar acquisition and resident exposure were correlated (R^2^ = 0.528, *p* < 0.001).

### 3.4. Effect of Age and Sex on Transmission

The potential effect of age on transmission was first tested by comparing the prevalence of infection amongst participants exposed as residents since childhood (<16 years of age; *n* = 196) to adults (>25 years of age; *n* = 45) that were exposed for a similar period (<16 years). No statistical differences were detected in the crude proportion of scar+ve or LST+ve (χ^2^_(1)_ < 1.87, *p* > 0.172). However, controlling for the significant location × exposure interaction term, the adjusted probabilities of being LST+ve or ACL scar+ve were significantly attributed to increasing age, beyond just length of exposure (z > 2.67, *p* < 0.008). For each year of increase in age, the risk of LST conversion increases by about 1.9% (IRR = 1.019 [95% C.I. 1.0068, 1.0312]), and the risk of presenting an ACL scar increases by about 2.2% (IRR = 1.022 [95% C.I. 1.006, 1.038]). Based on the same model, no differences were detected in crude or adjusted risks of infection between sexes (z < 1.69, *p* > 0.091). Nor did the subject recall of lesion appearance differ between sexes, either in median age or in age distributions (z < 1.140, *p* > 0.253).

### 3.5. Concordance between LST and Scars

**Figure 2:**
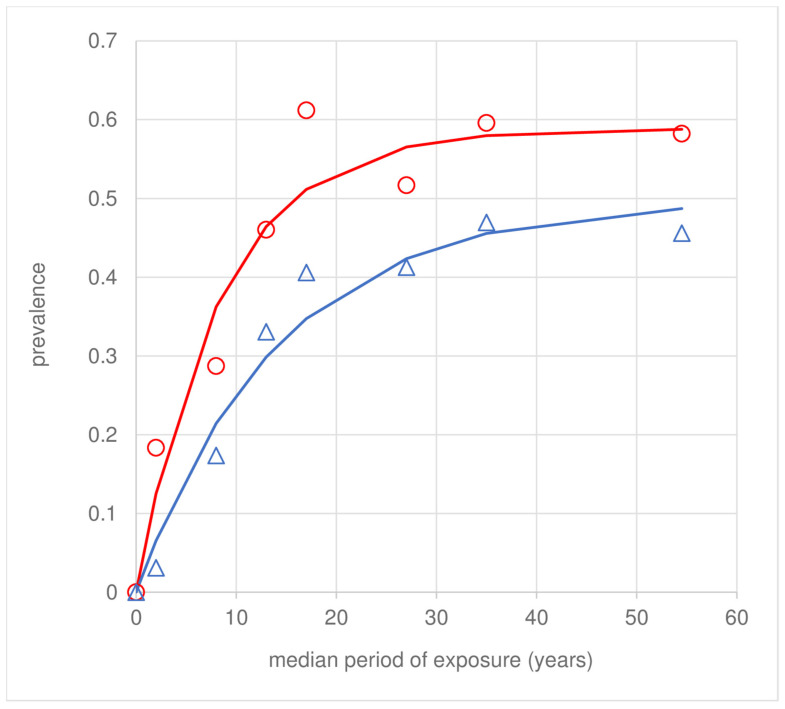
Regional prevalence of human LST+ves (red) and ACL scars (blue) with cumulative exposure. Aggregated data across sample foci (symbols) were fitted to the incidence–recovery model (Equation (1)) by maximum likelihood (line fits). The values of λ and ρ are shown in Table 2 and Table 3.

Most participants (84.3% [424/503]) showed concordance between LST and ACL scar status (Table 4), and the exposure-prevalence profiles showed similar trajectories (Figure 2). Of 166 scar+ves, 158 (95.2%) were LST+ve. Of 229 LST+ves, 31.0% (71/229) did not present any ACL scars. Only 4.8% (8/166) of individuals were LST-ve despite presenting ACL scars; of these, 6/8 individuals were infected as children (<18 years). Participants with signs of exposure/infection (LST+ve and/or scar+ve) reported longer periods of residency than participants that were LST-ve and scar-ve (z = 3.03, *p* = 0.002) (Table 4), adjusted for the significant infection status × location interaction term. By contrast, no differences were observed in exposure periods between LST+ve that did or did not present an ACL scar (z < 1.15, *p* > 0.250).

## 4. Rodents

### 4.1. Rodent Sample

Rodent infection rates were calculated by extracting demographic and diagnostic records from the CMR database of 603 marked individuals of eight wild and synanthropic rodent species (re)captured on 11,051 occasions [6]. Data for two rodent species (*Oligoryzomys nigripes* and *Cerradomys subflavus*) were omitted from the current analyses as only two and four individuals, respectively, were captured (all qPCR negative), generating too few data to estimate species-specific infection rates. Thus, the analyses in the present study were based on records of 596 marked individuals from six rodent species.

### 4.2. Rodent Infection

Rodent PCR/qPCR+ve prevalence was 0.43 (256/596); none of the animals sampled presented skin lesions or any other apparent clinical signs of rodent leishmaniasis. The period prevalences were highest in *Nectomys squamipes* (z > −3.26, *p* < 0.001) and *Holochillus sciureus* (z > −2.01, *p* < 0.045) compared to the other species (Table 5). The crude incidence of new infections amongst PCR/qPCR-ve animals at first capture tended to be higher in *Necromys lasiurus* than in other species. Fitting the number of new infections amongst the PCR/qPCR negative animals to Equation (2) produced the highest FOI λ/month value for *N. squamipes* (0.467) and the lowest for *Rattus rattus* (0.145). However, the variance around these estimates was broad for all species (z < 1.09, *p* > 0.275). The mean of FOI values across species was λ = 0.265/month. The species-specific median days to infection from the time of 1st capture for PCR/qPCR-ve animals were approximately 1.5–3 months (Table 5).

**Figure 3:**
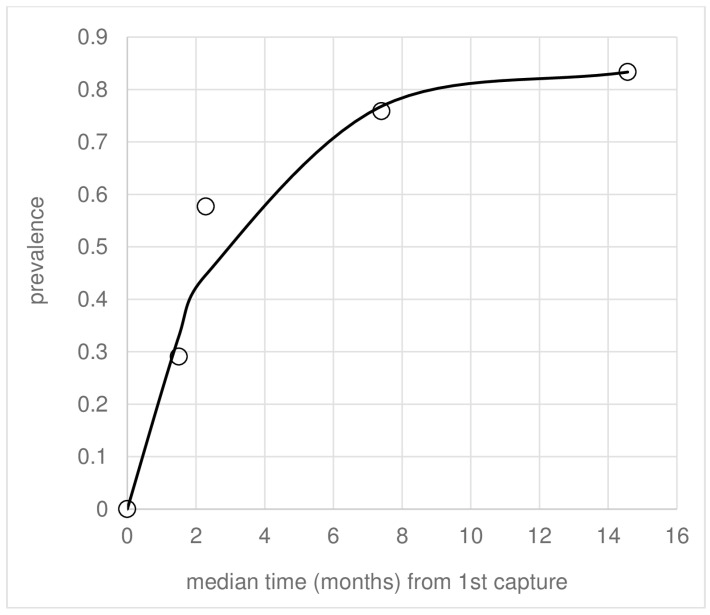
Age-prevalence data (symbols) for the cohort of rodents classified as juvenile (JJ) at first capture fitted to the incidence–recovery model (Equation (1)), giving the best fit (line) and estimates of λ and ρ by maximum likelihood. The age at first capture was assumed to be 6 weeks (corresponding to zero on the *X*-axis).

Considering the rodent population as a single reservoir guild, 79 conversions to qPCR+ve occurred amongst 126 individuals in 137.5 days, giving λ = 0.222/month (SD 0.1833) (Equation (2)). Focusing only on young animals classified as juveniles (JJ) at 1st capture, the age-prevalence data fitted to Equation (1) gave λ = 0.279 (SD: 0.050) by maximum likelihood, reaching an asymptote prevalence of ~0.8 (Figure 3). Using Equation (2), 10 of 14 juvenile rodents converted in a median 191-day follow-up, giving a similar result λ = 0.203/month (SD. 0.0945) and a mean time to infection from first capture of ~3.5 months (Figure 3; Table 6). Equivalent data for subadults (JA) and adults (AA) as categorised at 1st capture gave similar FOI values to those for juveniles, as well as median times to infection (z < 1.54, *p* > 0.124) (Table 6). The similarities in age-class λ estimates were reflected in the crude incidence data (Table 6).

### 4.3. Loss of Infection

From the longitudinal data of each rodent captured and tested on at least four independent occasions, individuals were defined as “recovered” from first-time infection if they proved PCR/qPCR-ve on at least two subsequent consecutive capture rounds and with no further sampling rounds diagnosed as PCR/qPCR+ve thereafter. Based on this criteria, 7/32 (0.219) infected animals recovered in a mean follow-up time of 5.86 months, giving ρ = 0.042/month (Equation (2)). Recovered animals reverted to -ve in a median of 3.61 months (range: 87–397) from the first observed time of infection. All seven recoveries were observed in *N. squamipes*, giving a species-specific value of 7/23 in 5.38 follow-up months and ρ = 0.068/month. These values of ρ are not significantly different from those (ρ = 0.053/month) resulting from the maximum likelihood fit of the age-prevalence data of juveniles shown in Figure 3.

## 5. The Association between Human and Rodent *Leishmania* Infection

Twenty-one human participants were residents of eight rural households in the Amaraji foci where the CMR epidemiological study of the rodent community was conducted. The median distance from each house to each of the other houses was 825 metres (IQR: 525–1700; range: 37–2900). Residents in 5/8 houses presented signs of *Leishmania* exposure; about half (10/21) tested LST+ve; and a third (7/21) presented ACL scars (Table 7). These proportions mirrored values in the wider community (Table 2 and Table 3). Households and associated adjacent plantations were locations previously identified as likely hotspots of human exposure [6] and where data were available for 731 diagnostic (re)captures of rodents (Table 7). PCR/qPCR prevalences included 0.30 (13/44) of *R. rattus* individuals on 27% of 48 (re)capture events in the immediate vicinity of households (peridomestic), and 0.48 (203/423) individual rodents of 4–6 species on 46% (312/683) of (re)capture events in household plantations (Table 7). The aggregated data for rodents showed a weak but positive correlation with the spatially associated household infection prevalences (LST: R^2^ = 0.323; ACL scars: R^2^ = 0.318).

### Xenodiagnosis

Xenodiagnosis was performed on 32 individual rodents on 35 occasions when (re)captured in the plantations of 6 of the 8 homesteads (Table 7). These included *N. squamipes, Ne. lasiurus*, *R. rattus*, and *Oxymycterus dasytrichus*. Two additional *R. rattus* were xenodiagnosed on a single occasion, each captured in two peridomestic trapping sites, and three individuals (one *A. cursor* and two *N. squamipes*) on four occasions when trapped in Atlantic Forest. All individuals, on all occasions, proved potentially infectious to the local sand fly vector, *Ny. whitmani*.

## 6. Discussion

Loss of LST responsiveness or ACL scars is suggested by dampening of the infection prevalence profiles with increasing cumulative exposure or age (Figure 1 and Figure 2). From Equation (1), the annual estimates for the study population were ρ = 0.05 (LST) (Table 2) and ρ = 0.04 (ACL scars) (Table 3), respectively. The term ρ, labelled here as recovery, can be attributed to non-mutually exclusive causes, including (i) true loss of LST responsiveness or ACL scars; (ii) higher loss of infected compared to uninfected individuals through death or emigration with increasing age or exposure; and (iii) a significantly lower exposure/FoI with increasing age, or in time.

ACL is not usually a fatal disease in mammalian hosts. In humans, a significant rate of ACL scar loss seems unlikely; nonetheless, the fraction of infections classified as clinical may be underestimated if there is a significant loss of scars or the rate of scar loss exceeds the loss of LST responsiveness. The values of ρ for scar loss and LST+ve reversions in this study were similar. An overestimate of the recovery rate could be attributed to the accumulation of resistant individuals in older age—or longer exposure—groups. On the other hand, LST recovery rates could be underestimated in areas with high or continuous reinfection rates, effectively masking recovery. Further studies are warranted to test these possibilities.

Regarding (iii), previous human epidemiological surveys in Amaraji [11] report similar LST+ve prevalences (34% in 1990, 46% in 1996) as in the current study in 2014. By contrast, the annual FOI λ = 0.092–0.107 in the previous surveys was higher than in the present study (λ = 0.03 in Amaraji; λ = 0.07 for the study population as a whole). Coupled with higher clinical to subclinical infection ratios of 4.6:1 (240/52) and 7.3:1 (29/4) compared to 2.3:1 (166/71) in the current study, this suggests a plausible shift over time in the pathogenicity of circulating *L.* (*V*.) *braziliensis* strains. Historically, the incidence was higher, which is clearly seen in the differences in CL/MCL case numbers notified to the MoH, showing substantial reductions and downward trends in recent years, both in Pernambuco state and, more generally, in Brazil [11,12,13]. During the 3-year study period (2012–2014), only 8 cases were reported in Amaraji (average of 281 cases per annum in Pernambuco), compared to 28–122 reported annual cases in Amaraji (average of 590 cases p.a. in Pernambuco) between 1988 and 1997 [11]. These data indicate that the recovery values ρ measured here were not due to a significantly lower exposure/FoI with increasing time. Nor do comparisons of the rodent age-class incidence values (Table 6) suggest short-term changes in λ during this period of study. Alternative explanations for the decline in reported ACL case numbers might include improvements in nutritional status and consequential disease resistance in the study populations, notwithstanding the similar downward trends in case numbers reported across Brazil. The accuracy of clinical diagnosis by local health workers and MoH notification systems may have improved over time. However, this is a well-known disease in this region by virtue of the historically high incidence of ACL/MCL cases and recognisable clinical signs. Molecular confirmation of clinical aetiologies is not routine; hence, increased accuracy is unlikely to have significantly affected reported case numbers.

The cumulative prevalence of LST+ve participants was 0.455 (229/503), and the cumulative proportion that presented ACL scars was 0.319 (167/524). By solving the incidence–recovery model parameters λ and ρ (Equation (1)), the annual FoI of LST+ve conversions for the study population as a whole λ = 0.07 (95% CI. 0.047, 0.093) was higher than of ACL scar acquisition λ = 0.04 (95% C.I.: 0.024, 0.046). This might be expected, as 31.0% (71/229) of LST+ves did not present ACL scars, indicative of subclinical infections. The possibility that some infections were misclassified due to unusually long incubation periods is not supported by our data. Of the 63 individuals who reported active lesions within 12 months prior to the survey, 58 were LST+ve at the time of the survey. And of the 166 participants presenting ACL scars, 158 (95.2%) were LST+ve at the time of the survey, confirming the high sensitivity of LST to detect clinical infections and not dissimilar to reported sensitivities of 90–93% in the previous LST surveys in Amaraji, which were higher than in other ACL regions [11]. The incubation period from ACL disease to LST+ve conversion is usually weeks and certainly <12 months [14]. We did not rely on participant-age recall to estimate the FOI of scar acquisition due to the likely recall bias towards younger ages.

A positive LST response is associated with ACL-specific CMI, providing protection against subsequent *Leishmania* clinical infections. However, CMI may not be lifelong nor fully protective, as evidenced by the reported frequency of reinfections, recurrent or reactivation of human ACL [15,16]; both low [11] and high [17] rates of LST+ve to LST-ve reversions are reported. This study did not follow up individuals to measure such reversions directly. However, the low prevalence of 0.048 (8/166) LST-ves amongst ACL scar+ves suggests that LST+ve responses to infection were the norm and that LST reversions were minimal. These eight individuals reported active lesions 1–16 years prior to the survey, suggesting that some may never have mounted a lasting LST+ve response. Failure to mount an initial specific immune response to *L.* (*V.*) *braziliensis* or other cutaneous leishmaniasis aetiological species is usually associated with a patient’s anergic state and/or decrease in CMI [18].

High numbers of synanthropic and wild rodents spatially associated with the Amaraji homesteads were infected, including 30% of 44 *R. rattus* individuals captured in the peridomestic setting and 48% of 423 individuals of 4–6 rodent species captured in adjacent household plantations (Table 7). Of the 34 individual rodents in these locations that were also xenodiagnosed, all were positive and potentially infectious. The human infection prevalences in these households (0.476 LST+ve and 0.333 ACL scar+ve) were not dissimilar to rates in the wider study population. Despite the limited number of households included in the CMR study area, household-level human and spatially associated rodent infection rates showed a weak but positive correlation (R^2^ = 0.32–0.32). Considering the rodent infection rates, species abundance, densities, and their potential infectiousness to the sand fly vector, we previously identified household plantations as the locations with the highest relative risk of transmission to humans [6]. The absence of significant differences between sexes in human LST and scar infection rates, and the relatively small increases in risk associated with increasing age, confirm previous conclusions that specific work-related activities are not a key driver of human transmission risk in this region [11]. Human residents of all ages and sexes routinely tended their cash crops in the plantations, all located within 20–40 m of their houses, and at sand fly biting times [19].

Rodent recovery rates were ρ = 0.053 and 0.068/month from Equations (1) and (2), respectively. The latter was based on an arbitrary definition of recovery applied to the longitudinal data, in this case identifying potential recoveries in 7/23 individuals, all *N. squamipes*, which was the most abundant and (re)captured species in the CMR study [6]. Rodent populations naturally infected with *Leishmania* typically support asymptomatic subclinical infections [20,21,22], with exceptions [23]. In this study, none of the animals presented any apparent clinical signs [6]. Thus, *Leishmania*-induced mortality seems unlikely. Rodent blood and skin tissues were tested by qPCR, revealing generally low geometric mean parasitaemia loads (11–97 per 0.2 μL) [6]. The possibility that some low parasite loads fell below the qPCR detection limit might help explain the apparent loss of PCR/qPCR signal, resulting in intermittent switches from qPCR+ve to qPCR-ve and vice versa between consecutive captures. This could lead to a false quantity of reversions. We acknowledge that the number of follow-up recaptures was limited, excluding options to test alternative definitions of recovery. The fact that these species of rodents could recover from clinical infection is supported by data from colonies of *N. squamipes*, *Ne. lasiurus*, and *R. rattus*, sourced from the Amaraji foci, that were experimentally infected with a high dose (5.5 × 10^6^/mL) *L.* (*V.*) *braziliensis* inoculum, resulting in ear and/or tail base skin lesions in 50% of 26 animals [2]. All these animals spontaneously healed within ~3 weeks of lesion appearance with no further signs of disease. Spontaneous recovery from rodent *Leishmania* natural infection is also seen in the wild [24].

The multiple FOI estimates for rodents were consistent and substantially higher (λ = 0.203–0.279/month) relative to those for humans (this study) or during previous LST surveys in Amaraji [11]. They are also higher than λ = 0.10–0.12/month reported for crab-eating foxes and λ = 0.11–0.26/month for domestic dogs, hosts of *L. infantum* in Brazil [25,26]. One current estimate (λ = 0.279) was derived from assuming a priori that juveniles (JJ) were, on average, 6 weeks old at first capture. Aging live animals is not an exact science, and the FOI estimate is sensitive to parameter *a* (Equation (1)). However, after varying *a* between 4 and 8 weeks, we confirmed that it did not alter the mean λ estimate substantially.

Of note in the current study is that 33% of the 186 individual rodents captured on more than one occasion were captured in multiple geographically distinct ecotypes or locations, indicating a network/corridor of rodent infection and potential infectiousness connecting the domestic and peridomestic (plantations) settings. Recaptures of these individuals also extended into the fragmented Atlantic Forest patches, where the general rodent infection prevalence was 0.460. Overall, 79% of these roaming rodents tested PCR/qPCR positive, and all of those tested were xenopositive [6]. Moreover, at least 25 of these individuals were recaptured in the plantations of 1–2 neighbouring household plantations, 10 animals of which proved infected at all captures, and 4/4 were xenopositive and potentially infectious. The inter-household plantation recapture locations were a median distance of 170 m apart (IQR: 170–650; range: 37–2900). Most (48.1%) of these excursions were by *N. squamipes,* one key member of the Amaraji reservoir species guild [2,6]. These distances were not dissimilar to home length distances reported for the same species in Atlantic Forest in Rio de Janeiro State: mean 165 m (*n* = 29 individuals) as measured by CMR, and 305–1055 m (*n* = 4) as measured by radio telemetry [27].

*L.* (*V*.) *braziliensis* parasites are detected in human symptomatic and asymptomatic infections [28], and household case clustering of ACL is reported [29], suggesting that they could contribute to onward transmission. However, the high FOI values and potential infectiousness amongst the rodent guild in this setting strongly suggest that transmission to humans results predominantly from spill-over infections from rodents. The dominant zymodeme (Z74) in Amaraji [30] is detected in *N. squamipes*, *Ny. whitmani*, and humans, further supporting the notion of spill-over transmission. Shared single nucleotide polymorphisms (SNPs) obtained from the paired genome sequences of ten previously characterised *L. (V.) braziliensis* zymodemes from Amaraji [31] fall into three genetic groups, the larger composed of four of these zymodemes. So far, no zymodeme or molecular group has been associated with specific pathologies or an exclusive host or vector species. More strains need to be analysed to confirm if any specific host or clinical condition is associated with a genotype or genotypic group. Domestic dogs support *L.* (*V*.) *braziliensis* infection, though its role in transmission is debated in the absence of xenodiagnoses [32,33]. One limitation of the xenodiagnosis procedure in this study is that post-exposure, blood-fed sand flies were immediately preserved in ethanol for DNA extraction and qPCR, i.e., prior to the extrinsic incubation period. The key difficulty was keeping blood-fed *Ny. whitmani* sand flies alive for metacyclic promastigote development. Hence, we cautiously label xenopositive animals in this study as potentially infectious.

*Ny. whitmani* is partially anthropophilic in feeding preference [34,35], a highly competent vector [36], largely exophilic/exophagic, and active year-round [19,34,37]. It is considered a forest species, though it has adapted to anthropogenic habitats throughout its geographical range [34,38]. The Atlantic Forest region has undergone extensive habitat alterations through deforestation and the creation of agricultural pastures and commercial plantations. Undoubtedly, small rodent populations with “fast” life history traits (high reproduction/population turnover rates, adaptive strategists) outcompeted and expanded in these habitats to become predominant reservoirs of *L.* (*V*.) *braziliensis*. Under certain conditions in accordance with the dilution effect hypothesis [39], such anthropogenically modified reductions in biodiversity can increase the relative abundance of competent reservoir species *versus* sink host species, with a consequential increase in transmission risk to humans. Such patterns are empirically implicated for Amazonian leishmaniasis, though, as the authors warn, different mechanisms may simultaneously dilute and amplify vector-borne transmission [40].

In conclusion, the human exposure/infection rates with *L.* (*V.*) *braziliensis* in the Zona de Mata region of Pernambuco State appear to be marginally lower compared to historical records. However, prevalences remain high despite a significant decrease in the number of notified ACL cases to the MoH. The infection rates in synanthropic and wild rodent populations in and around monitored households and associated plantations are high, and it is proposed that humans are at greatest risk of spill-over infection from a guild of infected rodent species. A significant proportion of these rodents enable an infection corridor linking forests, household plantations, and houses where the vector and rodent reservoirs are abundant. The relatively low FOI in humans compared to rodents is likely due to the interplay between demographic, behavioural, physical, and immunological parameters governing parasite–vector–host contact rates, which requires further investigation. Follow-on studies to monitor human and rodent infection dynamics in more recent years would also be informative. Possible interpretations of infection/scar/LST recovery are discussed.

## Figures and Tables

**Table 1:** Human study population sample.

Study Population/Foci	Numbers LST Tested (with Age/Exposure Data)	Numbers Examined for Scars (with Time of Occurrence Data)	Numbers with Records for Both LST and ACL Scars; (with Exposure Occurrence Data)	Median Age in Years (Q1, Q3)	M/F Sex Ratio
Amaraji	70	70	70	24 (5.6, 58.0)	0.75
Moreno	170	180	170	15 (3.0, 61.0)	1.22
Vicência	263 (261)	274 (272)	263 (261)	23 (6.7, 60.4)	1.62
All	503 (501)	524 (522)	503 (501)	20 (5.0, 60.0)	1.32

**Table 2:** Crude prevalence, instantaneous incidence (Force of Infection λ/year), and recovery (ρ/year) rates in three human study foci. Values of λ and ρ were estimated by fitting the LST prevalence data to the incidence–recovery model (Equation (1)).

Study Population/Location Foci	Number LST+ve/Number Tested (Proportion)	λ (95% C.L.)	ρ (95% C.L.)	Median (Q1, Q3) Period of Residential Exposure in Years
Amaraji	24/70 (0.343)	0.03 (0.003, 0.054)	0.02 (0.000, 0.084)	15 (2.0, 41.4)
Moreno	57/170 (0.335)	0.04 (0.015, 0.061)	0.04 (0.000, 0.090)	12 (2.0, 54.9)
Vicência	148/263 (0.563)	0.15 (0.055, 0.243)	0.11 (0.028, 0.186)	20 (5.0, 60.0)
All combined	229/503 (0.455)	0.07 (0.048, 0.093)	0.05 (0.020, 0.078)	15 (3.0, 57.0)

**Table 3:** Crude prevalence of ACL scars, instantaneous incidence (Force of Infection λ/year), recovery (ρ/year) rates, and median recall age of the ACL skin lesion leading to the ACL scar. Values of λ and ρ were calculated by fitting the ACL scar prevalence to the incidence–recovery model (Equation (1)).

Location	Number *Leishmania* Scar Positive/Number Tested (Proportion)	λ (95% C.L.)	ρ (95% C.L.)	Median Age (IQR) at the Time of Active Lesion Appearance in Years
Amaraji	15/70 (0.214)	0.02 (0.006, 0.024)	-	20 (1.5, 46.0)
Moreno	22/180 (0.122)	0.02 (0.001, 0.031)	0.04 (0.000, 0.147)	20 (7.0, 62.0)
Vicência	130/274 (0.475)	0.07 (0.032, 0.116)	0.06 (0.010, 0.114)	13 (3.3, 50.7)
Total	167/524 (0.319)	0.04 (0.024, 0.046)	0.04 (0.011, 0.059)	14 (4.0, 52.0)

**Table 4:** Association between LST and ACL scar status at the time of survey of the human study population and reported period of residence at the time of ACL lesion appearance.

Infection Status (*n*)	Median (Q1, Q3) Period of Residential Exposure in Years Prior to Appearance of ACL Active Lesion
LST+ve scar+ve (158)	20 (8.0, 60.0)
LST+ve scar-ve (71)	15 (2.0, 57.0)
LST-ve scar+ve (8)	16 (6.0, 55.0)
LST-ve scar-ve (266)	13 (2.0, 54.7)

**Table 5:** Period prevalence and infection rates were calculated from the longitudinal diagnostic records of (re)captured individuals of the six rodent species.

Rodent Species	Period Prevalence (Number Infected Animals/Total Tested)	Incidence/Month (Number of New Infections/Uninfected at First Capture)	Force of Infection λ/Month (SD) ^1^	Median (IQR) Days to Infection from First Capture
*Akodon cursor*	0.125 (4/32)	0.147 (2/3)	0.24 (0.195)	70 (26–124)
*Holochillus sciureus*	0.425 (17/40)	0.139 (5/8)	0.22 (0.124)	41 (41–63)
*Necromys lasiurus*	0.337 (28/83)	0.283 (10/15)	0.47 (0.208)	46 (41–50)
*Nectomys squamipes*	0.615 (150/244)	0.130 (43/62)	0.22 (0.055)	52 (32–114)
*Oxymycterus dasytrichus*	0.327 (16/49)	0.190 (8/13)	0.29 (0.138)	93 (44–141)
*Rattus rattus*	0.277 (41/148)	0.110 (11/25)	0.15 (0.053)	53 (41–133)
Totals ^a^/mean (95% C.I.s) ^b^	0.430 (256/596) ^a^	0.141 (79/126) ^a^	0.27 (0.049, 0.481) ^b^	59 (21–97) ^b^

^1^ Coefficients were estimated from longitudinal follow-up data using Equation (2). Only animals whose PCR/qPCR was negative at first capture were included in this calculation. Values on the last line represent (a) the proportion of the total counts; or (b) the mean and 95% C.I.s of the species-specific proportions (b).

**Table 6:** Infection incidence by rodent age class. FOI λ estimates were calculated from longitudinal follow-up diagnostic records (Equation (2)). Individuals were assigned to an age class at first capture.

Age-Class ^1^ Assigned at First Capture	Crude Incidence/Month (Number of Incident Cases/Number Uninfected at First Capture) ^2^	FOI λ/Month (SD) ^2^	Median (IQR) Days to Infection from First Capture
JJ	0.116 (10/14)	0.203 (0.0945)	114 (53–135)
JA	0.123 (28/37)	0.231 (0.0756)	47 (31–120)
AA	0.166 (41/75)	0.240 (0.0524)	50 (41–77)

^1^ Age class categorised at first capture: JJ—juveniles; JA—subadults; AA—adults. ^2^ λ coefficients reflect the numbers of uninfected rodents at first captured that developed infection over the mean follow-up time, estimated by Equation (2).

**Table 7:** Infection prevalences in households and in rodents captured in spatially associated peridomestic and plantation trap locations. Nd: no data.

	Humans: Number +ve/n Tested ^1^		Rodents: Number +ve/n Tested (Proportion) ^2^			
			Peridomestic Locations		Plantation Locations	
Home-stead ID	LST	ACL scars	Individual rodents	Capture events	Individual rodents	Capture events
1	5/7	3/7	Nd	Nd	32/63 (0.51)	49/119 (0.41)
2	1/4	0/4	1/3 (0.33)	1/3 (0.33)	6/17 (0.35)	6/20 (0.30)
3	0/3	0/3	4/26 (0.15)	4/30 (0.13)	55/116 (0.47)	98/201 (0.49)
4	2/2	2/2	1/3 (0.33)	1/3 (0.33)	17/33 (0.52)	20/39 (0.51)
5	0/1	0/1	0/1 (0.00)	0/1 (0.00)	31/56 (0.55)	55/110 (0.50)
6	1/1	1/1	6/10 (0.60)	6/10 (0.60)	48/86 (0.56)	66/125 (0.53)
7 ^b^	0/1	0/1	Nd	Nd	6/32 (0.19)	7/39 (0.18)
8 ^b^	1/2	1/2	1/1 (1.00)	1/1 (1.00)	8/20 (0.40)	11/30 (0.37)
sum	10/21	7/21	13/44 (0.30)	13/48 (0.27)	203/423 (0.48)	312/683 (0.46)

^1^ Infection is indicated by LST+ve and/or ACL scars attributed to *Leishmania* infection. ^2^ Infection is indicated by qPCR and/or PCR of rodent tissue samples as described [6]. ^b^ Xenodiagnosis was performed on rodents captured at all homesteads except for these two.

## Data Availability

Data is contained within the supplementary material.

## Supplementary Materials

The following supporting information can be downloaded at: https://www.mdpi.com/article/10.3390/pathogens12121395/s1, Table S1. Leishmania Skin Test (LST) and American Cutaneous Leishmaniasis scar (ACL) status, age, sex, and residence in study foci of human participants.
