# Supplementary material for: Incidence of Human and Free-Ranging Wild Rodent Infections with Leishmania (Viannia) braziliensis, Aetiological Agent of Cutaneous Leishmaniasis
Source: Pathogens. 2023 Nov 28;12(12):1395. doi: 10.3390/pathogens12121395 (PMC10746019; doi:10.3390/pathogens12121395)
Supplement: Supplementary file 1 [file pathogens-12-01395-s001.zip › pathogens-2701078-supplementary.pdf]

Table S1. Leishmania Skin Test (LST) and American Cutaneous Leishmaniasis scar (ACL) status, age, sex, and residence in study foci of human participants.

local	age (yrs)	exposure (yrs)	LST	ACL scar	sex
Amaraji	40	40	1	1	1
Amaraji	44	44	0	0	2
Amaraji	8	8	0	0	1
Amaraji	11	11	1	0	2
Amaraji	34	24	0	0	2
Amaraji	6	6	0	0	2
Amaraji	8	8	0	0	1
Amaraji	58	27	1	1	2
Amaraji	17	15	0	0	1
Amaraji	14	1	0	0	1
Amaraji	13	13	0	0	2
Amaraji	14	14	0	0	1
Amaraji	26	26	1	0	2
Amaraji	15	15	0	0	2
Amaraji	62	29	1	1	2
Amaraji	28	13	0	0	1
Amaraji	38	11	0	0	2
Amaraji	39	28	1	1	2
Amaraji	12	12	0	0	2
Amaraji	26	25	1	0	1
Amaraji	6	6	0	0	2
Amaraji	32	32	0	0	2
Amaraji	8	8	0	0	2
Amaraji	37	20	0	0	2
Amaraji	41	19	1	1	1
Amaraji	57	19	1	0	2
Amaraji	6	6	0	0	1
Amaraji	23	19	0	0	1
Amaraji	58	50	1	0	2
Amaraji	37	32	1	0	2
Amaraji	21	12	0	0	1
Amaraji	37	18	0	0	2
Amaraji	15	15	0	0	1
Amaraji	18	18	0	0	1
Amaraji	24	4	0	0	1
Amaraji	34	12	0	0	2
Amaraji	10	1	0	0	1
Amaraji	17	13	0	0	1
Amaraji	49	22	1	1	1
Amaraji	14	14	0	0	2
Amaraji	17	17	1	1	1
Amaraji	25	25	1	1	1
Amaraji	49	20	0	0	2
Amaraji	43	20	1	0	1
Amaraji	27	22	0	0	2
Amaraji	29	29	0	0	1
Amaraji	67	26	1	1	2

Amaraji	13	13	1	1	2
Amaraji	24	18	1	0	2
Amaraji	34	15	1	1	2
Amaraji	16	16	0	0	2
Amaraji	35	35	0	0	2
Amaraji	8	8	0	0	2
Amaraji	43	43	0	0	1
Amaraji	12	12	1	0	2
Amaraji	38	8	0	0	2
Amaraji	14	7	0	0	1
Amaraji	6	6	0	0	2
Amaraji	19	14	0	0	1
Amaraji	23	23	1	1	2
Amaraji	39	10	0	0	2
Amaraji	34	2	0	0	2
Amaraji	45	38	1	1	1
Amaraji	5	5	1	1	2
Amaraji	9	9	0	0	2
Amaraji	2	2	0	0	2
Amaraji	56	30	1	1	1
Amaraji	5	5	0	0	1
Amaraji	21	16	0	0	1
Amaraji	16	2	0	0	1
Moreno	61	16	1	0	2
Moreno	10	10	0	0	1
Moreno	33	15	0	0	1
Moreno	4	4	0	0	2
Moreno	51	15	0	0	1
Moreno	1	1	0	0	2
Moreno	28	28	0	0	2
Moreno	9	9	0	0	1
Moreno	4	4	0	0	2
Moreno	31	31	0	0	1
Moreno	5	5	0	0	1
Moreno	58	58	0	0	1
Moreno	2	2	0	0	1
Moreno	20	20	0	0	1
Moreno	6	6	0	0	2
Moreno	9	9	0	0	1
Moreno	10	10	1	1	1
Moreno	16	16	0	0	2
Moreno	6	6	0	0	1
Moreno	62	52	0	0	2
Moreno	8	8	0	0	2
Moreno	14	14	0	0	1
Moreno	10	10	0	0	2
Moreno	66	15	1	1	1
Moreno	32	4	0	0	1
Moreno	14	14	0	0	2
Moreno	14	14	1	1	1

Moreno	14	14		0	2
Moreno	15	7		0	2
Moreno	8	8	0	0	1
Moreno	40	13	0	0	1
Moreno	14	14		0	2
Moreno	42	42	1	0	1
Moreno	13	13	0	0	2
Moreno	14	14	1	1	1
Moreno	12	12	0	0	1
Moreno	32	10	1	0	1
Moreno	36	1	0	0	2
Moreno	16	9	0	0	1
Moreno	42	42	0	0	1
Moreno	5	5	0	0	2
Moreno	10	10	0	0	2
Moreno	14	6	0	0	2
Moreno	30	6	1	0	1
Moreno	2	2	0	0	2
Moreno	28	15	1	0	1
Moreno	12	12	0	0	1
Moreno	11	11	0	0	1
Moreno	42	15	1	1	1
Moreno	41	15	1	0	2
Moreno	31	15	1	1	1
Moreno	54	14	0	0	1
Moreno	12	12	0	0	2
Moreno	10	10	0	0	2
Moreno	7	7	0	0	2
Moreno	6	6	0	0	1
Moreno	14	14	0	0	1
Moreno	35	17	1	1	2
Moreno	43	15	0	0	1
Moreno	3	1	0	0	2
Moreno	23	2	0	0	1
Moreno	52	12	1	0	1
Moreno	13	13	0	0	1
Moreno	43	3		0	1
Moreno	9	5	1	0	1
Moreno	29	17	0	0	1
Moreno	54	20	1	1	1
Moreno	56	56	0	0	2
Moreno	45	10	0	0	2
Moreno	38	10	1	0	1
Moreno	16	7	1	0	1
Moreno	18	7	1	0	1
Moreno	3	3	0	0	2
Moreno	24	7	0	0	1
Moreno	9	9	1	0	2
Moreno	14	10	0	0	2
Moreno	6	6	0	0	1

Moreno	3	3	0	0	2
Moreno	2	2	0	0	2
Moreno	22	7	0	0	1
Moreno	48	48	1	0	1
Moreno	28	7	1	1	1
Moreno	5	5	1	0	1
Moreno	10	10	0	0	2
Moreno	5	5	0	0	2
Moreno	30	30	0	0	2
Moreno	11	11	0	0	2
Moreno	13	13	1	0	1
Moreno	12	12	0	0	1
Moreno	14	14		0	1
Moreno	54	54	0	0	2
Moreno	12	12	0	0	1
Moreno	49	17	0	0	2
Moreno	8	8	0	0	1
Moreno	11	11	1	0	2
Moreno	36	17	0	0	1
Moreno	9	9	0	0	1
Moreno	11	11	0	0	1
Moreno	39	39	1	1	1
Moreno	54	17	0	0	2
Moreno	61	15	0	0	1
Moreno	13	13	1	0	2
Moreno	11	11	0	0	2
Moreno	60	17	1	0	2
Moreno	51	17	1	1	2
Moreno	16	16	0	0	1
Moreno	13	13	0	0	2
Moreno	16	16	0	0	1
Moreno	45	29	0	0	1
Moreno	59	13	1	0	2
Moreno	19	1	1	0	1
Moreno	14	14	0	0	1
Moreno	10	10	0	0	2
Moreno	3	3	0	0	1
Moreno	29	29	0	0	1
Moreno	22	7	1	0	1
Moreno	5	5	1	0	2
Moreno	52	2		0	1
Moreno	24	2		0	2
Moreno	3	3	0	0	2
Moreno	7	7	1	0	2
Moreno	33	33	0	0	1
Moreno	52	52	1	0	1
Moreno	10	10	0	0	2
Moreno	10	10	0	0	1
Moreno	57	57	1	0	1
Moreno	60	60	1	0	2

Moreno	62	16	1	0	2
Moreno	58	17	0	1	1
Moreno	62	62	1	1	2
Moreno	36	1	0	0	1
Moreno	30	12	1	1	2
Moreno	9	1	0	0	2
Moreno	34	2	1	0	1
Moreno	34	15	0	0	2
Moreno	17	16	0	0	1
Moreno	15	2	1	0	1
Moreno	9	2	1	0	2
Moreno	10	10	0	0	2
Moreno	9	9	0	0	2
Moreno	28	28	0	0	1
Moreno	15	15	0	0	2
Moreno	9	9	0	0	2
Moreno	49	17	1	0	1
Moreno	8	7	0	0	2
Moreno	1	1		0	2
Moreno	6	6		0	1
Moreno	41	12	0	0	1
Moreno	10	10	0	0	1
Moreno	11	11	1	1	2
Moreno	7	7	0	0	2
Moreno	15	12	0	0	2
Moreno	20	20	0	0	1
Moreno	22	20	1	1	2
Moreno	14	14	1	1	1
Moreno	52	8	0	0	1
Moreno	14	14	1	1	2
Moreno	12	12	1	1	1
Moreno	33	17	1	0	1
Moreno	14	14	0	0	1
Moreno	6	6	0	0	1
Moreno	14	14		0	1
Moreno	16	16	1	1	2
Moreno	24	24	0	0	1
Moreno	56	56	0	0	1
Moreno	5	5	0	0	1
Moreno	8	8	0	0	1
Moreno	12	12	1	1	2
Moreno	6	6	0	0	2
Moreno	6	6	0	0	2
Moreno	2	2	1	0	2
Moreno	12	12	0	0	1
Moreno	2	2	0	0	2
Moreno	61	59	0	0	1
Moreno	15	15	0	0	2
Moreno	62	6	0	0	1
Moreno	40	36	1	0	1

Moreno	28	6	1	0	2
Moreno	19	19	1	1	2
Moreno	79	77	1	0	2
Vicencia	60	50	0	1	2
Vicencia	55	55	0	1	1
Vicencia	53	53	1	1	1
Vicencia	67	67	0	0	1
Vicencia	71	61	1	1	1
Vicencia	57	57	1	1	1
Vicencia	44	44	1	1	1
Vicencia	65	65	0	0	1
Vicencia	60	60	0	0	2
Vicencia	10	10	0	1	1
Vicencia	9	9	0	0	2
Vicencia	56	55	1	0	2
Vicencia	33	33	1	1	1
Vicencia	57	57	1	1	1
Vicencia	30	30	1	1	1
Vicencia	23	23	1	1	1
Vicencia	20	20	0	0	1
Vicencia	47	10	0	0	1
Vicencia	25	25	0	0	1
Vicencia	21	21	0	0	1
Vicencia	26	26	1	1	1
Vicencia	28	28	0	0	1
Vicencia	8	8	0	0	2
Vicencia	10	10	1	1	2
Vicencia	27	27	0	0	1
Vicencia	31	31	0	0	1
Vicencia	8	8	1	1	2
Vicencia	7	7	0	1	1
Vicencia	10	10	1	0	2
Vicencia	29	29	1	0	1
Vicencia	35	35	1	1	1
Vicencia	32	32	0	0	1
Vicencia	5	5	0	0	1
Vicencia	14	14	1	1	1
Vicencia	13	13	1	1	2
Vicencia	1	1	0	0	2
Vicencia	33	33	1	1	1
Vicencia	7	7	1	0	1
Vicencia	17	12	1	1	1
Vicencia	3	3	0	0	2
Vicencia	59	59	1	1	1
Vicencia	33	33	1	1	1
Vicencia	32	32	0	0	1
Vicencia	52	40	1	1	1
Vicencia	4	4	0	0	2
Vicencia	13	13	1	0	1
Vicencia	14	14	1	1	2

Vicencia	13	13	1	1	2
Vicencia	8	8	0	0	1
Vicencia	11	8	1	1	2
Vicencia	38	38	1	1	2
Vicencia	16	16	1	1	1
Vicencia			0	0	1
Vicencia	19	19	1	1	1
Vicencia	13	13	1	1	1
Vicencia	23	23	1	1	1
Vicencia	33	33	1	1	1
Vicencia	22	22	1	1	1
Vicencia	12	12	0	0	2
Vicencia	27	27	1	1	1
Vicencia	17	17	1	1	1
Vicencia	32	32	1	0	1
Vicencia	40	40	0	0	1
Vicencia	15	15	0	0	1
Vicencia	42	42	1	1	1
Vicencia	14	14	0	0	2
Vicencia	14	14	1	0	1
Vicencia	21	21	0	0	1
Vicencia	58	58	1	1	1
Vicencia	16	16	1	1	1
Vicencia	11	11	1	1	1
Vicencia	21	21	1	1	1
Vicencia	19	19	1	1	2
Vicencia	12	12	1	1	2
Vicencia	23	23	0	0	1
Vicencia	21	21	0	0	2
Vicencia	11	11	0	0	1
Vicencia	7	7	0	0	1
Vicencia	45	45	1	1	1
Vicencia	13	13	0	0	2
Vicencia	15	15	1	1	1
Vicencia	15	15	1	1	1
Vicencia	14	14	0	0	1
Vicencia	43	43	0	0	
Vicencia	69	69	1	1	1
Vicencia	61	61	1	1	2
Vicencia	60	60	1	1	1
Vicencia	62	62	0	0	1
Vicencia	43	43	1	1	1
Vicencia	6	6	0	0	1
Vicencia	37	37	0	0	2
Vicencia	17	17	1	0	1
Vicencia	16	16	1	0	2
Vicencia	35	35	0	0	1
Vicencia	18	18	0	0	1
Vicencia	34	34	0	0	1
Vicencia	32	3	0	0	2

Vicencia	61	61	0	0	2
Vicencia	48	48	0	0	2
Vicencia	48	48	0	0	1
Vicencia	30	20	1	1	1
Vicencia	11	11	1	1	2
Vicencia	55	55	0	0	1
Vicencia	30	30	1	1	1
Vicencia	8	8	1	1	1
Vicencia	13	13	1	0	2
Vicencia	42	11	1	1	2
Vicencia	28	25	1	0	1
Vicencia	11	11	1	1	2
Vicencia	34	34	0	0	1
Vicencia	37	7	1	1	1
Vicencia	35	35	1	1	2
Vicencia	38	15	1	1	1
Vicencia	63	63	1	1	2
Vicencia	9	9	0	0	1
Vicencia	25	1	0	0	1
Vicencia	37	15	0	0	1
Vicencia	19	19	1	1	2
Vicencia	37	37	0	0	2
Vicencia	8	8	0	0	2
Vicencia	34	34	0	0	1
Vicencia	10	10	0	0	2
Vicencia	43	43	1	1	1
Vicencia	18	18	0	0	2
Vicencia	14	14	1	1	2
Vicencia	28	28	0	0	2
Vicencia	19	19	1	1	1
Vicencia	18	18	1	0	2
Vicencia	16	16	0	0	2
Vicencia	52	52	1	1	2
Vicencia	28	28	1	1	2
Vicencia	13	3	0	0	2
Vicencia	15	15	1	1	1
Vicencia	16	16	1	1	1
Vicencia	30	30		0	2
Vicencia	17	17		0	2
Vicencia	14	14	0	0	1
Vicencia	12	12	0	0	1
Vicencia	14	14	0	1	1
Vicencia	38	38	0	0	1
Vicencia	69	69	1	1	2
Vicencia	27	27	1	1	2
Vicencia	19	19	0	0	1
Vicencia	28	28	0	0	1
Vicencia	21	21	1	1	1
Vicencia	18	18	1	1	1
Vicencia	27	27	1	1	1

Vicencia	11	11	1	1	2
Vicencia	12	12	1	0	2
Vicencia	10	10	0	0	2
Vicencia	33	30	1	0	2
Vicencia	49	49	1	1	2
Vicencia	40	40	1	0	2
Vicencia	25	25	1	1	1
Vicencia	5	5	1	1	1
Vicencia	9	9	1	1	1
Vicencia	14	14	1	1	2
Vicencia	12	12	1	0	2
Vicencia	27	27	0	0	1
Vicencia	59	59	1	1	1
Vicencia	17	17	1	1	1
Vicencia	14	14	1	0	2
Vicencia	35	35	1	1	1
Vicencia	9	9	1	0	1
Vicencia	61	61	0	0	1
Vicencia	41	22	0	0	1
Vicencia	34	34	1	1	2
Vicencia	37	37	1	1	2
Vicencia	44	44	1	1	2
Vicencia	44	44	0	0	1
Vicencia	38	38	0	0	1
Vicencia	12	12	0	0	1
Vicencia	6	6	0	1	1
Vicencia	23	23	0	0	1
Vicencia	20	20	0	0	1
Vicencia	26	26	1	1	1
Vicencia	33	33	1	1	2
Vicencia	17	17	1	1	2
Vicencia	17	17	1	1	2
Vicencia	17	17	1	0	2
Vicencia	65	25	0	1	2
Vicencia	63	63	0	0	1
Vicencia	43	43	0	0	1
Vicencia	32	32		0	1
Vicencia	16	16	1	1	2
Vicencia	57	57	1	0	1
Vicencia	16	16	0	0	2
Vicencia	34	34	0	0	1
Vicencia	9	9	0	0	1
Vicencia	21	21	0	0	1
Vicencia	17	17	1	1	2
Vicencia	48	48	1	1	2
Vicencia	39	39	1	1	1
Vicencia	18	18	1	0	2
Vicencia	27	27	0	0	2
Vicencia	30	30	0	0	1
Vicencia	28	28	0	0	1

Vicencia	54	54	0	0	1
Vicencia	27	27	1	1	2
Vicencia	9	9	1	1	1
Vicencia	10	10		0	1
Vicencia	10	10	1	1	2
Vicencia	37	37	1	1	2
Vicencia	41	41	1	1	1
Vicencia	26	26		0	1
Vicencia	15	15	0	0	1
Vicencia	60	60	0	0	1
Vicencia	31	31	1	1	1
Vicencia	33	33	1	1	1
Vicencia	45	45		0	2
Vicencia	20	20	1	1	1
Vicencia	9	9	1	1	2
Vicencia			1	1	2
Vicencia	9	9	0	0	2
Vicencia	38	38	1	1	1
Vicencia	39	39		0	1
Vicencia	31	31	0	0	1
Vicencia	3	3	0	0	1
Vicencia	7	7	0	0	1
Vicencia	35	35	1	1	1
Vicencia	8	8	1	1	2
Vicencia	14	14	1	1	1
Vicencia	64	10	1	1	2
Vicencia	46	46	1	1	2
Vicencia	37	37	0	0	2
Vicencia	54	54	1	1	2
Vicencia	33	33	1	1	1
Vicencia	4	4	0	0	1
Vicencia	5	5	0	0	1
Vicencia	5	5	0	0	1
Vicencia	11	11	1	1	1
Vicencia	8	8	1	0	1
Vicencia	11	11	0	0	2
Vicencia	15	15	1	0	2
Vicencia	53	53	1	0	1
Vicencia	39	39	1	0	2
Vicencia	12	12	0	0	1
Vicencia	11	11	1	1	2
Vicencia	12	12	1	1	1
Vicencia	13	12	1	1	1
Vicencia	15	15	0	0	1
Vicencia	14	14	0	0	1
Vicencia	9	9	1	1	2
Vicencia	13	13		1	1
Vicencia	13	13	0	0	2
Vicencia	16	16	1	1	1
Vicencia	18	18	1	1	1

Vicencia	19	19	1	0	
Vicencia	30	30	1	1	2
Vicencia	57	57	1	1	2
Vicencia	60	30	1	1	2
Vicencia	18	18	0	0	1
Vicencia	5	5	0	0	1
Vicencia	2	2	0	0	1
Vicencia	46	46	1	1	1
Vicencia	9	9		0	2
Vicencia	41	41	0	0	1
Vicencia	15	15	0	0	2
Vicencia	30	30		0	2
Vicencia	23	23	0	0	1
Vicencia	9	9	0	0	1
Vicencia	30	30	0	0	2
Vicencia	56	56	1	1	1
Vicencia	13	13	1	1	1
Vicencia	16	16		0	1
Vicencia	9	9	0	0	2
Vicencia	22	22	0	0	2
Vicencia	60	60	1	1	2
Vicencia	28	28	0	0	2
Vicencia	39	39	1	1	2
Vicencia	38	3	0	0	1
Vicencia	11	11	0	0	2
Vicencia	8	3	0	0	1
Vicencia	12	12	1	1	1
